# Improved mutation tagging with gene identifiers applied to membrane protein stability prediction

**DOI:** 10.1186/1471-2105-10-S8-S3

**Published:** 2009-08-27

**Authors:** Rainer Winnenburg, Conrad Plake, Michael Schroeder

**Affiliations:** 1 Biotechnology Center, Technische Universität Dresden, Tatzberg 47-49, Dresden, 01307, Germany

## Abstract

**Background:**

The automated retrieval and integration of information about protein point mutations in combination with structure, domain and interaction data from literature and databases promises to be a valuable approach to study structure-function relationships in biomedical data sets.

**Results:**

We developed a rule- and regular expression-based protein point mutation retrieval pipeline for PubMed abstracts, which shows an F-measure of 87% for the mutation retrieval task on a benchmark dataset. In order to link mutations to their proteins, we utilize a named entity recognition algorithm for the identification of gene names co-occurring in the abstract, and establish links based on sequence checks. Vice versa, we could show that gene recognition improved from 77% to 91% F-measure when considering mutation information given in the text. To demonstrate practical relevance, we utilize mutation information from text to evaluate a novel solvation energy based model for the prediction of stabilizing regions in membrane proteins. For five G protein-coupled receptors we identified 35 relevant single mutations and associated phenotypes, of which none had been annotated in the UniProt or PDB database. In 71% reported phenotypes were in compliance with the model predictions, supporting a relation between mutations and stability issues in membrane proteins.

**Conclusion:**

We present a reliable approach for the retrieval of protein mutations from PubMed abstracts for any set of genes or proteins of interest. We further demonstrate how amino acid substitution information from text can be utilized for protein structure stability studies on the basis of a novel energy model.

## Background

Proteins carry out most cellular functions as they are acting as building blocks for structures, enzymes, and gene regulators, and are involved in cell mobility and communication [1]. Proteins may interact briefly with each other in an enzymatic reaction, or for a long time to form part of a protein complex. The interactions between proteins are of central importance for almost all processes in living cells, and are described by numerous distinct pathways in databases such as KEGG [2]. Malfunctions or alterations in such pathways can be the cause of many diseases, when for instance the biosynthesis of involved proteins is repressed or proteins are not interacting the way they should. The latter can be due to structural changes in one of the interacting proteins, caused by point mutations, i.e. single wild type amino acid substitutions. Indeed, it is already well known that such mutations are the cause of many hereditary diseases. Thus the large-scale analysis of point mutation data in combination with information about protein interactions, protein structure, and disease pathogenesis might facilitate the study of still unresolved phenotypes and diseases. Despite the availability of numerous biomedical data collections, valuable information about mutation-phenotype associations is still hidden in non-structured text in the biomedical literature. This knowledge can be extracted by text mining, stored in a homogeneous data store, and integrated with already available data from suitable databases. Combining all data, new hypotheses can be formulated, such as the prediction of phenotypic effects induced by mutations.

Genomic variation data have already been collected for many years. Single nucleotide polymorphisms (SNPs), which make up about 90% of all human genetic variation and occur every 100 to 300 bases along the 3-billion-base human genome [3], are available as large collections. Single amino acid polymorphisms (SAPs) are often manually extracted from literature and curated into databases, originating from wet lab experiments. Additionally, some structures of such mutations may be revealed in crystallography experiments and might eventually end up as distinct structures in the Protein Database PDB. Of particular interest is the identification of mutations which have a strong influence on the stability of proteins. Therefore, the biomedical literature can be systematically searched for information about mutation-phenotype associations by text mining, which may lead to new insights beyond information in existing databases. For the text mined data it is additionally possible to weight or prioritize information according to publication date, the involved authors, and journals. Consideration of such meta data can be relevant for detecting that an already published assumption has been proven wrong in a more recent publication, or for determining whether a protein just recently attracted interest or if the information is already available for years. Furthermore, it is possible to receive a more detailed view on a protein's characteristics, for example, if a certain interaction only takes place under specific conditions, or if an interaction is prevented by the conformational change of a protein domain triggered by a point mutation.

### Databases

Data on mutations have been collected for years, for numerous species and by different organizations for diverse purposes. There are many efforts to cope with the data, which is being made available in a growing number of databases. The Human Genome Variation society [4] promotes the collection, documentation and free distribution of genomic variation information. New mutation databases are reported in the journal Human Mutation on a regular basis. There are manually curated databases like OMIM [5], UniProt Knowledgebase [6,7], and general central repositories like the Human Gene Mutation Database HGMD (now part of BIOBASE) [8], Universal Mutation Database [9], Human Genome Variation Database [10], or MutDB [11]. Besides these central repositories, there are small specialized databases, such as the infevers autoinflammatory mutation online registry [12], the GPCR NaVa database for natural variants in human G protein-coupled receptors [13], or the Pompe disease mutation database with 107 sequence variants [14]. Table 1 compares available mutation databases in terms of their scope and information content.

**Table 1:** Mutation databases: Most of available mutation databases focus on mutations from human, or specific protein families (e.g. G protein-coupled receptors). Some lack well-defined information on mutant phenotypes and only few link to interaction data. Half of the databases also contain data retrieved by text mining methods.

	**Species**	**SNPs**	**Protein mutations**	**Diseases**	**Phenotypes**	**Interactions**	**Text Mining**
OMIM [5]	human	+	+	+	+	+	-
HGMD [8]	human	+	+	+	+	-	+
UniProtKB [6]	various	-	+	-	+	-	+
HGVbase [10]	human	+	-	+	+	-	-
HapMap [41]	human	+	-	-	-	-	-
dbSNP [42]	44	+	+	-	+	-	-
MutDB [11]	human	+	+	-	-	+	-
GPCRDB [43]	18	-	+	-	+	-	-
GPCR NaVa [13]	human	+	+	+	+	-	-
CoagMDB [44]	human	+	+	-	+	-	+
OSIRIS [45]	human	+	-	+	-	-	+
PolySearch [46]	various	+	+	+	-	+	+

In contrast, unpublished SNPs normally make their way into large locus specific data repositories. Since August 2006, there is a wiki based approach SNPedia  in contrast to classical databases collecting information on variations in human DNA.

### Text mining

Despite the availability of numerous biomedical data collections, valuable information about mutation-phenotype associations is still hidden in non-structured text in the biomedical literature. Hence, text mining methods are implemented to automatically retrieve these data from the 18 millions of referenced articles in PubMed [15-19]. Text mining aims to generate new hypotheses through the automatic extraction and integration of information spread over several natural language texts. One of the key prerequisites for finding new *facts *(e.g. *interactions *or *mutations*) is the named entity recognition (NER) in text [20,21], the assignment of a class to an entity (e.g. *protein*), as well as a preferred term or identifier, in case an entry in a database, such as *UniProt*, or a controlled vocabulary like the *Gene Ontology (GO) *[22] exists. For the task of named entity recognition usually a dictionary is used, which contains a list of all known entity names of a class (e.g. human proteins) including synonyms. For the recognition of patterns (e.g. database identifiers like *NM_12345*) regular expression can be defined. For the analysis of whole sentences, *Natural language processing (NLP) *techniques are used, which aim to understand text on a syntactic and semantic level. This approach is often paired with systems which are based on a set of manually defined *rules *or which make use of (semi-)supervised *machine learning *algorithms.

In recent years, there have been diverse examples for the successful application of text mining to the mutation retrieval task. Early examples are the automatic extraction of mutations from Medline and cross-validation with OMIM [23], and mining OMIM for phenotypic and genetic information to gain insights into complex diseases [24]. More recently, a concept recognition system based on regular expressions was applied on mutation mining task [25]. GraB for the automatic extraction of protein point mutations using a graph bigram association [26] was reported to reliably find gene-mutation associations in full text. For identifying gene-specific variations in biomedical text, the ProMiner system developed for the recognition and normalization of gene and protein names was integrated with a conditional random field (CRF)-based recognition system [27]. As an answer to the diverse approaches developed over the past years, a framework for the systematic analysis of mutation extraction systems was proposed [28].

A growing number of groups are working on protein mutations and their involvement in diseases. A recent overview is given at [29]. Kanagasabai et al. [30] developed mSTRAP (Mutation extraction and STRucture Annotation Pipeline), for mining mutation annotations from full-text biomedical literature, which they subsequently used for protein structure annotation and visualization. Worth et al. [31] use structure prediction to analyse the effects of non-synonymous single nucleotide polymorphisms (nsSNPs) with regard to diseases. Focusing on Alzheimer's disease, Erdogmus et al. [32] developed MuGeX to extract mutation-gene pairs, with estimated 91.3% recall, and precision at 88.9%. Lage et al. [33] realized a human phenome-interactome network of protein complexes implicated in genetic disorders by integrating quality-controlled interactions of human proteins with a validated, computationally derived phenotype similarity score.

We compared the above mentioned mutation extraction approaches with regard to their strengths and weaknesses. MutationFinder is still used as a reference system for the pure mutation extraction task, although it does not distinguish between mutations on the DNA and protein level, and does not support grounding to genes. MuGeX finds textual descriptions of mutations and distinguishes between DNA and protein mutations, but their mutation grounding relies only on proximity and does not consider sequence information. The mutation grounding approach used in mStrap considers sequence information, but allows only mutation-protein pairs that co-occur in one sentence and the mutation extraction approach relies on simple regular expressions. Finally, GraB is a successful approach which implements the grounding and disambiguation techniques discussed above, but might be computationally too expensive for large data sets. Towards the development of an automated system for the interpretation of structure-function relations in the context of genetic variability data, we chose to design our own protein mutation retrieval system. We aim at a system, which identifies and grounds protein mutations based on sequence information and proximity at a high recall. On the other hand we need a flexible system, that can be applied to diverse biomedical questions and has moderate computational requirements.

## Methods

As we have motivated above, novel gene-disease associations or the influence of mutations on protein-protein interactions can be discovered through combination of data from literature and databases. Hence, we designed a generic mutation centred approach that can be applied to any kind of genetic data for answering disease-centred questions. As a prerequisite, we consider available high quality data on protein point mutations from curated databases and from peer-reviewed literature. For the latter, we present a flexible approach for both the specific and high-throughput retrieval of mutations. In detail, the following tasks have to be performed: (1) Identify genes/proteins in abstracts. (2) From this subset of abstracts consider only those which additionally contain information about mutations. (3) Propose potential protein-mutation pairs. (4) Filter proposed pairs by sequence checks. (5) Utilize this information for the refinement of the original gene/protein identifier.

### Entity recognition

#### Gene normalization

This module allows for the automated named entity recognition of genes and proteins. Our approach performs gene name disambiguation by using background knowledge to match a gene with its context against the text as a whole [34]. A gene's context contains information on Gene Ontology annotations, functions, tissues, diseases etc. extracted from the databases Entrez Gene and UniProt. A comparison of gene contexts against the text gives a ranking of candidate identifiers and the top ranked identifier is taken if it scores above a defined threshold. This approach has been recently extended for inter-species gene normalization and achieves 81% success rate on a mixed dataset of 13 species [35].

#### Mutation tagging

We implemented an entity recognition algorithm (*MutationTagger*) to automatically extract protein point mutation mentions from PubMed abstracts. Wild-type and mutant amino acid, as well as the sequence position of the substitution are extracted by means of both a set of regular expressions for pattern recognition of 1 or 3-letter-notations (e.g. *E312A *or *Glu(312) → Ala*), and rules for the more complex identification of textual mutation descriptions (e.g. *Glu312 was replaced with alanine*). Problems concerning the full text representations (detecting the correct sequence position of the mutated residue and unraveling enumerations) have been addressed by additional extraction algorithms and the implementation of a sequence check. An evaluation of our method on the test data from MutationFinder [36] showed comparable success rates of 88% F-measure for mutation mention extraction (see Table 2).

**Table 2:** Mutation retrieval task: Evaluation of precision (P), recall (R), and F-measure (F) on a benchmark set provided with the MutationFinder algorithm. Our MutationTagger performs in general comparably to MutationFinder. Although MutationFinder shows a slightly better overall performance, in the high recall mode MutationTagger extracts more mutations, which is desirable for the subsequent grounding and gene normalization improvement task.

	MutationTagger_*precision*_			MutationTagger_*recall*_			MutationFinder		
			
	**P**	**R**	**F**	**P**	**R**	**F**	**P**	**R**	**F**
Extracted mutation mentions	0.970	0.807	0.881	0.914	**0.856**	0.883	**0.984**	0.819	**0.894**
Extracted mutations	0.956	0.786	0.863	0.870	**0.845**	0.857	**0.975**	0.807	**0.883**
Document retrieval	**1.0**	0.863	0.926	0.960	**0.912**	0.935	0.994	0.890	**0.939**

### Mutation grounding

In the process of recognizing mutations in text the direct association to specific proteins and genes remains a challenge. This is due to the fact that the abstracts of relevant publications typically mention more than one mutation or protein, respectively. Thus, a mutation – protein association purely based on their co-occurrence in one abstract is not sufficient, as the consideration of all possible combinations of mutations and proteins would result in a significant number of false positive predictions. The problem becomes even more evident, when considering that both gene and mutation tagging are imperfect, achieving a precision of 80 to 90% each.

We are aiming at an approach that disambiguates the relations of candidate mutations with proteins, and at the same time filters out false positives from the underlying mutation and gene recognition tasks. Approaches have already been developed, which apply a word distance metric for assigning a mutation to its nearest occurring protein term, which is error prone, as matching mutation and protein do not necessarily have to occur close to each other in the abstract or even in the same sentence. The statistical approach GraB is a tool for the automatic extraction of protein point mutations using a graph bigram association [26], achieving good results of up to 79% F-measure for mutation-protein association but alone would also not fulfil the second aspect of filtering out false positives.

#### Sequence checks

Mutations are commonly described as the substitution of a wild-type by a mutant amino acid at a given position. Our method compares the wild-type residue as described in a mutation mention with the UniProt/Swiss-Prot and PDB protein sequences for all candidate proteins. It is important to incorporate sequences from both repositories, as the sequence numbering can differ and it is not always evident from a publication's abstract, which the authors are referring to. To map UniProt IDs to PDB and vice versa, we used PDB cross-references in UniProtKB/Swiss-Prot  and the residue specific comparison between PDB and SwissProt sequences  as provided by Martin et al. [37]. Only associations between mutations and proteins with matching amino acids are considered, whereas the score of mismatches is set to 0. Matching pairs are scored based on their proximity, favouring pairs that co-occur in the same sentence. We assign the score to the gene – mutation pair, but also keep track of the particular Swiss-Prot and/or PDB sequence (including chain information) that matched to the mutation. In the case of a shift between Swiss-Prot and PDB sequences we calculate the correct numbering for the shifted sequence utilizing the mapping table by Martin et al. Through the consideration of both sequence and proximity information, for each mutation exactly one gene match is determined, even if more than one protein-mutation pair is possible.

### Annotation pipelines

**Figure 1:**
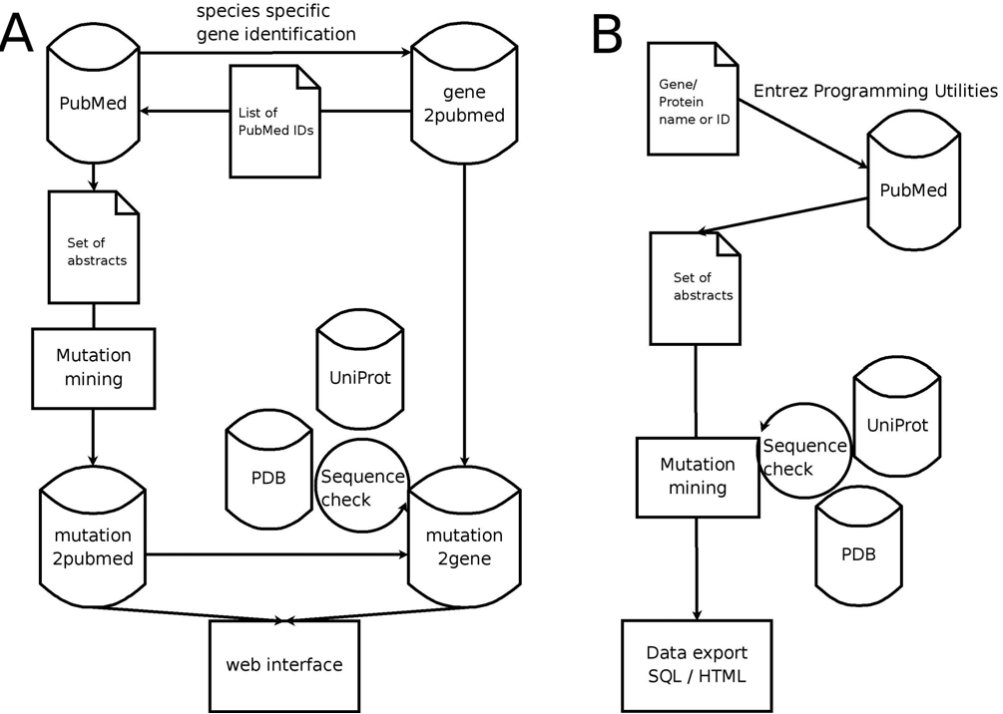
**Mutation retrieval workflow**. Workflow of mutation data retrieval with MutationTagger. **A: **PubMed IDs of abstracts mentioning proteins for given species are retrieved from a local database (gene2pubmed), which contains the results of our gene normalizing approach. Mutations are identified in the abstracts and stored (mutation2pubmed). The gene and mutation data is joined, filtered by sequence checks, and stored (mutation2gene). **B: **For a queried protein or gene relevant articles are retrieved from the Entrez database. Mutations are identified in the abstracts, sequence checks against the queried protein are performed, and the checked mutation data is exported to HTML or SQL.

The developed mutation retrieval pipeline can be accessed through two different interfaces (see Figure 1), which offer either a systematic or quick and flexible solution, dependent on the annotation task. The following approaches have been implemented:

#### Organism-centred approach (database)

All available mutations for a given organism will be retrieved in one literature screening and stored in the Mutation database. This approach relies on the large-scale identification of gene mentions in PubMed abstracts, which have to be compiled for organisms of interest prior to a mutation screening. As of now, gene mention data is available for Human, Mouse, Yeast, Rat, Fruit Fly, E. coli, A. thaliana, C. elegans, Zebrafish, and H. pilori. However, data for additional relevant organisms will be added on a regular basis in the near future.

#### Protein-centred approach (on-the-fly)

It is possible to retrieve relevant data for a single gene or a list of genes/proteins for any organism. For this purpose, relevant documents are obtained via a keyword searches from the PubMed library using the Entrez Programming Utilities. Like for the large-scale identification of gene mentions in PubMed abstracts in the organism-centred approach, the result is a set of abstracts, which is subsequently processed by the MutationTagger.

### Improvement of gene normalization

As described above, we defined the input set of documents for the organism-e mutation mining approach by scanning the whole PubMed database for abstracts mentioning at least one gene or protein of a pre-defined species. For this filtering step, we relied on the gene normalization techniques of our gene normalizer, which was applied to all PubMed abstracts in advance and has shown 85% F-measure for human genes and slightly lower for other species [35]. However, the gene normalization proposes only one identifier per gene mention, even if a set of different candidate identifiers was computed. According to internal ranking mechanisms, only the top scoring candidate is considered. This leads to a possible scenario, where in some cases the correct identifier is ranked lower and would be neglected for any subsequent data procession. In case of our mutation mining algorithm, we assume that some mutations cannot be associated to the correct protein, because the gene tagging task already failed.

On the other hand, it should be possible to improve the performance of both entity recognition techniques for genes and mutations by combining the results. The idea is to run both approaches with low precision thus receiving a high recall, associate all genes to all mutations, and then consider the intersection of all combinations that fit. Mutation and gene product are considered to be a valid pair, if the wild-type residues at the mutated position in the protein sequence and in the reported mutation match (as described in section *Sequence Checks*). For all proposed gene identifiers, protein sequences are obtained and checked for compliance with the reported wild type amino acid. The score of identifiers that show a match are increased, which might lead to a re-ranking of the identifiers for one gene entity. This could further improve the original gene normalization approach for candidate entities which are reported to show a mutation.

#### Example

**Figure 2:**
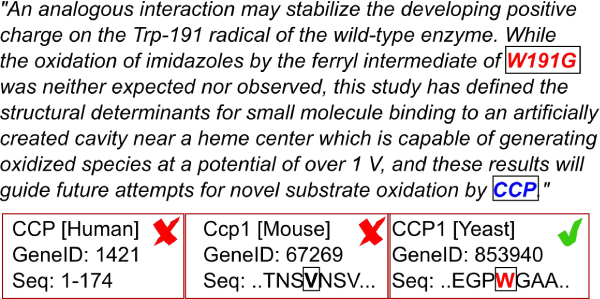
**Improvement of gene normalization**. Example for gene name normalization with the help of mutation mining. Initially, our gene normalizer proposed the human gene CCP as its context fits the text best (abstract not fully shown). However, when comparing the recognized mutation at position 191 with the sequences of all three candidates, only CCP in yeast contains the wild-type tryptophan at the specified position (PDB entry). After checking the full text of this publication, we found that CCP indeed refers to the gene in Saccharomyces cerevisiae.

As shown in Figure 2 our gene normalizer identified CCP (human crystallin, gamma D) with EntrezGene ID 1421 as the top candidate gene for abstract PMID 8142383. MutationTagger identified a replacement of tryptophan with glycine at position 191 as the only mutation mentioned in the paper. None of the protein sequences retrieved for human CCP showed a tryptophan residue at position 191, which means that this gene identifier was not supported by mutation information. However, besides human crystallin, there was also cytochrome-c peroxidase in yeast (EntrezGene ID 853940) proposed as an alternative identifier, which was ranked lower. As the product of this gene showed a tryptophan residue at position 191 (according to PDB sequencing) the score was increased making it the new top candidate. Indeed, manual curation of the corresponding literature confirmed, that the only gene mentioned in the abstract is cytochrome-c peroxidase in yeast.

## Results and discussion

### Mutation database

We are establishing a mutation database, which is intended to store all protein point mutations mentioned in PubMed abstracts for all organisms of interest. We realized an early version, comprising a MySQL database and web-interface to access the data. It is envisaged to apply the data on diverse biological problems, such as the detection of mutation centred gene-disease associations in human.

**Figure 3:**
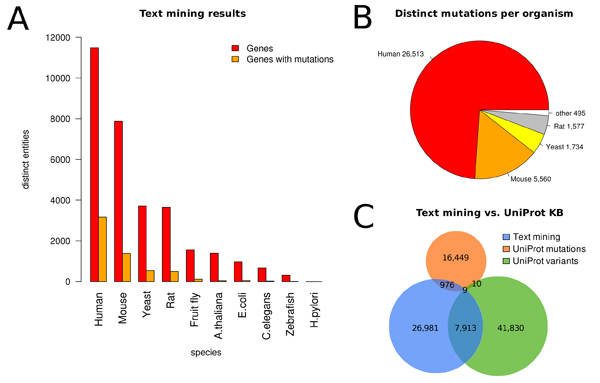
**Mutation database content**. Mutations and their genes extracted from text for ten model organisms. **A: **For each organism the number of distinct genes (red) and genes with mutations (orange) extracted from PubMed abstracts are shown. From the 6,000 distinct mutated genes found in total, more than half were human (3,170) which corresponds to 25% of all extracted human genes. **B: **The distribution of text mined mutations across organisms. More than 70% of all mutations reported in literature abstracts are from human. **C: **The Venn diagram shows text mined mutations (blue) in comparison to variant (green) and mutation (orange) annotations from UniProtKB as of version 1.47: information for additional 26,981 mutations was obtained through text mining.

To populate the database, in a first step the PubMed corpus is filtered for abstracts mentioning at least one gene or protein using the named entity recognition algorithm as described in section *Gene normalization*. Currently, data for 10 model organisms is available: Human, Mouse, Yeast, Rat, Fruit Fly, E. coli, A. thaliana, C. elegans, Zebrafish, and H. pilori. This led to a set of 1,564,124 abstracts proposing more than 3 millions of potential protein candidates. In the second step, the mutation extraction system is applied on this corpus and the retrieved information is transferred into the database. In total, 240,057 mutation mentions were found in 68,983 abstracts. Subsequently, for all candidate genes found in these abstracts, the corresponding sequences are obtained and checked for compliance with the wild type amino acid at the position of the mentioned mutation. Out of 451,474 potential protein – mutation pairs 106,360 are supported by sequence checks (59,991 if multiple mentions of the same mutation in one abstract are counted as one) in contrast to 345,114 (188,878) mutations which have not passed the sequence filter. In summary, from all 240,057 mutation mentions initially identified by the algorithm 100,681 (42%) could be supported by gene associations based on sequence check. These data were retrieved from 30,458 (44%) out of 68,983 abstracts in total. Figure 3 shows the content of the database for the different species and compares the text mining results with mutation data retrieved from UniProtKB. We made the mutation data for the ten model organisms available in GoGene [38] at .

### Improvement of gene normalization

We evaluated our approach on two different tasks: pure identification of a mutation in a text, and the identification of correct mutation-protein pairs. An evaluation of our method on the test data from MutationFinder [36] showed comparable success rates of 88% F-measure for pure mutation mention extraction (see Table 2). The test set comprises 508 abstracts which are manually annotated with point mutations. 183 out of 508 abstracts contain at least one mention of a point mutation. It should be noted that the annotation does not contain any information about genes or proteins. Our approach (MutationTagger in recall mode) found in 166 of 183 abstracts mutations, whereas 7 additional abstracts were wrongly predicted to contain mutation information. On the mutation level, 776 out of 907 mutation mentions were identified alongside 73 false positives. We found 33 correct mutations more than MutationFinder. The higher false positive rate is in regard to the mutation grounding task secondary, as we could observe that most of the falsely predicted mutations are discarded in the subsequent filter check. To assess the mutation grounding and gene name normalization improvement as motivated in the Methods section, we run our gene normalization approach on the 183 abstracts that contained mutations. We were able to identify gene mentions of any of the 10 supported species in 22 abstracts. It should be noted that the majority of the 183 abstracts contained genes from species that are not yet supported by our approach. In the initial run, the gene name normalizer identified in 17 of 22 abstracts (77%) the correct gene as the top ranked candidate. However, after the gene tagging refinement by applying the mutation-sequence filter to all candidate genes, in three more papers genes were identified correctly replacing the false top candidate. The following genes could be correctly identified after re-ranking: Cytochrome c peroxidase of yeast in PubMed abstract 8142383 (see also Figure 2), human TP53 in abstract 11254385, and human amylase alpha in abstract 15182367. This led to the correct normalization of all genes in 20 out of 22 (91%) abstracts. For the remaining two publications, the correct genes could not be identified, as they belong to species which are not yet supported by our system. The abstracts became part of our validation subset, as the gene normalizer falsely predicted mouse genes. However, these genes were subsequently not supported by the sequence checks and the proposed identifiers were ranked below the threshold. Showing no gene identification at all, these abstracts turned from the two "false positives" into "true negatives". The results on the test set indicate that our grounding approach performs reliably and can improve gene name normalization. In contrast to our approach of first performing sequence checks and using proximity as secondary information, most related grounding mechanisms do either not consider sequence information like MuGeX [32], or utilize it only as secondary information after proximity like mSTRAP [30]. In addition, we consider both UniProt and PDB sequences for sequence checks, as both are used by authors when describing mutations in the literature. Sequence checks are surprisingly specific already for single mutations, with increasing precision for double and triple mutants. However, the presence of some orthologous proteins in one abstract complicates the grounding of mutations.

### On-the-fly vs. database approach

We evaluated the results of the two approaches (database and on-the-fly) for human Aquaporin-1, as part of the stability analysis of protein membranes (see Section *Application*). Precision of the on-the-fly approach is expected to be lower, as the document retrieval part is relying on the more general free text queries through Entrez ESearch utility. We chose this approach to be independent of our gene normalization approach, which so far only supports 10 model organisms. Indeed, in comparison to the unique 20 mutations found by the organism-centred approach, 9 additional mutations were found querying for "(Chip28 OR Aquaporin-1) AND human". All of these additional mutations turned out to be false positives, actually appearing in Aquaporin-2 or 4. This supports the good precision of our gene normalization approach. We found out, that a slightly modified query "(Chip28 OR "Aquaporin-1") AND human" did not produce false positives and conclude, that query building might not work fully automated but needs human interaction. Similar problems could be observed, when short gene names or synonyms were part of queries and could be overcome by removing them from the query. On the other hand, this supports the good precision of our gene normalization approach.

### Application

#### Predicting effects of mutations based on sequence

Integral membrane proteins play an important role in all organisms, especially as transporters. Due to their striking importance, mutations in membrane proteins are known to be the cause of many hereditary diseases, such as cystic fibrosis, or retinitis pigmentosa. The reason are often conformational changes in proteins, which may lead to malfunction of a whole protein complex. Unfortunately, identified structures for membrane proteins are still rare. For this reason, we used a coarse grained model presented by [39] considering sequence information to assess the influence of mutations on protein structure.

The approach considers the solvation energy, which is based on the probability distribution for each amino acid within the integral part of a membrane protein to be facing the lipids of the membrane or the neighbouring proteins. The amino acid specific property "inside" or "outside" reflects the orientation of the amino acid side chains with respect to the centre of mass of the neighbouring residues. For a given mutation in an integral part of a membrane protein, the approach compares the solvation energies for wild-type and mutant residues. If the energies differ significantly, a destabilizing effect is predicted, especially if the energies are changing from negative to positive or vice versa.

To quantify the ability of this model to predict the influence of mutations on the stability of membrane proteins, we compared already examined and published effects of mutations with the predictions of the sequence based model. For this purpose, we screened the literature for single point mutations reported for five membrane proteins from the family of G protein-coupled receptors (bacteriorhodopsin and halorhodopsin from *Halobacterium salinarum*, bovine rhodopsin, Na+/H+ antiporter from *Escherichia coli*, and human aquaporin-1). As described in section *Results and Discussion*, *Protein-centred approach *and Figure 1B, articles relevant for these proteins were identified by searching PubMed via the NCBI Entrez Programming Utilities. Abstracts for each protein were queried by the protein and gene name including the synonyms as derived from the corresponding PDB/UniProt entry.

The MutationTagger was applied on these five sets of abstracts for the extraction of mutation information. The application of sequence checks brought the results down to a reasonable number of proposed mutations, which were presented as HTML documents and subsequently manually curated. In the manual curation phase, we only considered publications where a clear relationship between a single point mutation and stability or stability related function was described. Double or multiple mutations were not considered, if the determination of a direct relation between the reported effect and one of the mutations was not possible. If an appropriate mutation was found in the literature, we compared the solvation energies of both wild-type and mutant residues, which were calculate according to [39], to decide, if the mutation is stabilizing, slightly stabilizing, slightly destabilizing, or destabilizing.

#### Example

Mutation T93P for bovine rhodopsin was reported to lead to a conformational change of the protein [40]. Considering the two solvation energies of wild type Threonine (-0.66 a.u.) and mutant Proline (0.08 a.u.) a destabilizing effect can be predicted, although both amino acids are actually classified as neutral. Without the change of sign from - to +, only a slightly destabilizing effect would have been hypothesized.

#### Relevance

We were able to show the ability of our mutation mining approach to retrieve publications containing mutation information for given proteins at a good precision. Due to the quick and precise retrieval of mutation data we were able to assess the soundness of the coarse grained model for the prediction of stabilizing regions in membrane proteins. For any of these five membrane proteins, 25 out of 35 mutational effects reported in the literature correlate with the predictions based on the solvation energy (see Table 3). These cases suggest a relation between mutations and stability issues in membrane proteins. It should be noted that none of these mutations were annotated in the UniProt and PDB databases.

**Table 3:** Influence of mutations as predicted by the solvation energy based approach compared with the literature. For each of the five protein structures in the first column, we listed the text mined protein point mutations and the manually extracted effect of this mutation as reported in the literature. We compared these effects with the stability change prediction by our model and evaluated if these were in compliance. In 71% of the cases a change in function or stability is correlated with interaction energy.

**Protein name**	**Mutation in literature**	**Effect as reported in literature**	**Stability change**	**Compliance**
**Bacteriorhodopsin** PDB: 1brr UniProt: P02945	G113Q	destabilized	slightly destabilizing	yes
	G113L	destabilized	destabilizing	yes
	G116Q	destabilized	slightly destabilizing	yes
	G116L	destabilized	destabilizing	yes
	I117F	destabilized	slightly destabilizing	yes
	I117A	destabilized	stabilizing	no
	M145F	still active	destabilizing	no

**Halorhodopsin** PDB: 1e12 UniProt: P16102	H95A	destabilized	slightly destabilizing	yes
	H95R	destabilized	slightly stabilizing	no
	R108Q	not functional	slightly destabilizing	yes
	T203V	less active	destabilizing	yes

**Rhodospin** PDB: 1f88 UniProt: P02699	T93P	misfolded	destabilizing	yes
	T94I	night blindness	destabilizing	yes
	C110F	r. pigmentosa	destabilizing	yes
	C110Y	r. pigmentosa	destabilizing	yes
	C110A	r. pigmentosa	slightly destabilizing	yes
	E122Q	still active	slightly destabilizing	no
	E122D	still active	slightly destabilizing	no
	E122A		slightly destabilizing	
	E122R	no retinal binding	slightly destabilizing	yes
	C185A	wrong disulfide	slightly destabilizing	yes
	G188R	misfolding	slightly destabilizing	yes
	S186A	incr. activation energy	slightly destabilizing	yes
	C187Y	r. pigmentosa	destabilizing	yes
	C187A	r. pigmentosa	slightly destabilizing	yes
	N310C	less activity	slightly destabilizing	yes
	M317C	less activity	slightly destabilizing	yes

**Antiporter** PDB: 1zcd UniProt: P13738	A130C		slightly stabilizing	
	D133A	not functional	slightly destabilizing	yes
	H225P	less activity	destabilizing	yes
	H225C	less activity	none	no
	G303C	not functional	slightly destabilizing	yes

**Aquaporin** PDB: 1h6i UniProt: P29972	N42A	still active	slightly destabilizing	no
	A73M	not functional	slightly stabilizing	no
	Y186F	conduct water	slightly destabilizing	
	Y186A	no water conductance	stabilizing	
	Y186N	no water conductance	stabilizing	
	C189M	less activity	slightly stabilizing	no
	C189S	still active	slightly stabilizing	yes
	H209A	still active	slightly destabilizing	no

## Conclusion

We developed a rule- and regular expression-based approach that allows for the retrieval of protein point mutations from the whole PubMed database specifically for any given protein. This flexibility makes it a powerful tool for immediately finding relevant data for follow-up studies, as we showed in the application on five membrane proteins. In addition, MutationTagger can be utilized for the species-wide identification of mutations in proteins mentioned in PubMed. We started to set up a mutation database which allows for systematically querying mutation related information, and finding relevant literature for subsequent studies. The sequence checks applied on identified mutations and candidate proteins have been proven to be an efficient, yet not sufficient filter for mutation-protein associations. The filter shows good sensitivity but improvable specificity, especially regarding the species level. Furthermore, we were able to show that mutation information from literature can even further improve the quality of the gene normalization algorithm, which already showed very good results.

## Competing interests

The authors declare that they have no competing interests.

## Authors' contributions

RW implemented and applied the mutation extraction and grounding approach, and performed the analyses and manuscript writing. CP performed the gene normalization task and analysed the gene normalization improvement. MS supervised the development and analysis of the method, and producing the manuscript.
